# A systematic review and meta-analysis assessing antiretroviral therapy for treatment-experienced HIV adult patients using an optimized background therapy approach: is there evidence enough for a standardized third-line strategy?

**DOI:** 10.1186/s13643-022-02102-3

**Published:** 2022-11-17

**Authors:** Lucas Pitrez Mocellin, Patricia Klarmann Ziegelmann, Ricardo Kuchenbecker

**Affiliations:** 1 Universidade Federal do Pampa – Campus Uruguaiana, Administrative Building, Collective Room No. 2, BR 472, Km 592 – Caixa Postal 118, Uruguaiana, RS Brazil; 2 Statistics Department, Hospital de Clínicas de Porto Alegre, Universidade Federal Do Rio Grande Do Sul, Rua Ramiro Barcelos, Porto Alegre, RS 2350 Brazil; 3 Programa de Pós-Graduação Em Epidemiologia, Hospital de Clínicas de Porto Alegre, Universidade Federal Do Rio Grande Do Sul, Rua Ramiro Barcelos, Porto Alegre, RS 2350 Brazil

**Keywords:** HIV, Antiretroviral therapy, Multi-experienced HIV-1-infected patients, Third-line therapy, Systematic review and meta-analysis

## Abstract

**Background:**

The World Health Organization (WHO) has identified the need for evidence on third-line antiretroviral therapy (ART) for adults living with HIV/AIDS, given that some controversy remains as to the best combinations of ART for experienced HIV-1-infected patients. Therefore, we conducted a systematic review and meta-analysis to (i) assess the efficacy of third-line therapy for adults with HIV/AIDS based on randomized controlled trials (RCT) that adopted the “new antiretroviral (ARV) + optimized background therapy (OBT)” approach and (ii) address the key issues identified in WHO’s guidelines on the use of third-line therapy.

**Methods:**

MEDLINE, EMBASE, LILACS, ISI Web of Science, SCOPUS, and Cochrane Central Register of Controlled Trials were searched for RCTs assessing third-line ARV therapy that used an OBT approach between 1966 and 2015. Data was extracted using an Excel-structured datasheet based on the Consolidated Standards of Reporting Trials (CONSORT) recommendations. The primary outcome of this meta-analysis was the proportion of patients reaching undetectable HIV RNA levels (< 50 copies/mL) at 48 weeks of follow-up. Included studies were evaluated using the Cochrane’s Risk of Bias assessment tool. Summarized evidence was rated according to the GRADE approach.

**Results:**

Eighteen trials assessing 9 new ARV + OBT combinations defined as third-line HIV therapy provided the efficacy data: 7 phase IIb trials and 11 phase III trials. Four of the 18 trials provided extension data, thus resulting in 14 trials providing 48-week efficacy data. In the meta-analysis, considering the outcome regarding the proportion of patients with a viral load below 50 copies/ml at 48 weeks, 9 out of 14 trials demonstrated the superiority of the new combination being studied (risk difference = 0.18, 95% CI 0.13–0.23). The same analysis stratified by the number of fully active ARVs demonstrated a risk difference of 0.29 (95% CI 0.12–0.46), 0.28 (95% CI 0.17–0.38) and 0.17 (95% CI 0.10–0.24) respectively from zero, one, and two or more active drugs strata. Nine of the 18 trials were considered to have a high risk of bias.

**Conclusions:**

Efficacy results demonstrated that the groups of HIV-experienced patients receiving the new ARV + OBT were more likely to achieve viral suppression when compared to the control groups. However, most of these trials may be at a high risk of bias. Thus, there is still not enough evidence to stipulate which combinations are the most effective for therapeutic regimens that are to be used sequentially due to documented multi-resistance.

**Supplementary Information:**

The online version contains supplementary material available at 10.1186/s13643-022-02102-3.

## Introduction

Since 2010, the World Health Organization’s public health approach for the use of antiretroviral therapy (ART) to treat and prevent HIV infection has included the recommendation that national programs should develop policies for first-, second-, and third-line ART combinations [1]. The rationale of such approaches includes that of maximizing HIV treatment efficacy and safety by reducing the range of different ART combinations. However, clinical trials usually prioritize combinations of antiretroviral drugs to optimize individual treatment strategies for patients, without necessarily evaluating regimens that allow combinations with some capacity for generalization, allowing the definition of second and third-line regimens, despite efforts looking for alternatives like these [2, 3].

While antiretroviral (ARV) drugs have significantly reduced the number of people who present with resistance-related virological failure, a substantial number of individuals require combinations according to the viral resistance profile acquired during previous treatments, a condition that poses a challenge to the evaluation and recommendation of standardized second- and third-line regimens. The Joint United Nations Programme on HIV/AIDS (UNAIDS) has predicted 28.5 million PLHIV on ART worldwide in 2025, corresponding to 24.3 million on first-line therapy, 3.5 million on second-line therapy, and 0.6 million on third-line therapy [4]. And yet, 23 years after the introduction of the highly active antiretroviral therapy (HAART), third-line ART still entails considerable uncertainty due to the limited number of options available for these cases [5], especially given that there are few studies addressing the best ART combinations and the treatment impact on the progression of the disease and on AIDS-related mortality.

Hence, from a public health perspective, some controversy remains as to the best ART combinations for experienced HIV-1-infected patients [6–8]. Current guidelines simply recommend that previously failed regimens should be changed to new combined ART (cART) according to the results of past and current resistance tests. New cART should include a minimum of 2 and “preferably” [6, 7] or “ideally” 3 fully active drugs [8] chosen by genotype–phenotype assessment to be used in combination with optimized background therapy (OBT). It is worth emphasizing that the clinical recommendations of “preferably”, or “ideally” 3 fully active drugs chosen by genotype-phenotype assessment to be used in combination with OBT have remained unchanged in British and North American antiretroviral treatment guidelines for almost a decade [9–11]. Again, these recommendations, although based on the best available evidence, are still general guidelines, without, however, providing guidelines that can be characterized as combinations of second or third lines [9–11]. Another additional challenge is limited access to tests to assess HIV resistance to antiretroviral treatment. WHO does not currently endorse HIV drug resistance testing for individual patient management, a condition that reinforces the need for expansion of optional second and third-line regimens, such as those based on dolutegravir, for example, known to be associated with a greater barrier to the emergence of viral resistance such as way of enabling the public health approach to HIV treatment [12]. Salvage regimens are recommended with drugs such as darunavir/ritonavir (DRV/r), etravirine (ETV), dolutegravir (DTG), and raltegravir (RAL), containing regimens with or without previously used ARV [13]. Nevertheless, the WHO Guidelines characterized those recommendations as “conditional” (i.e., desirable effects of adherence to a recommendation probably outweigh the undesirable effects, but is low confident), and as having been based on studies that provided low-quality evidence. The WHO also pointed out that most of the studies used as the foundation for the guidelines had been conducted in limited settings, that is to say middle-to-high and high-income, and therefore, the transferability of this knowledge to lower-resourced settings is unclear [13].

Despite the lack of a clearly delineated statement of how third-line therapy should be implemented, WHO’s Guidelines recommend that national programs should develop policies for third-line therapies and that the corresponding approaches should optimize regimens using genotype profiles and the addition of new drugs with minimal risk of cross-resistance to previously used regimens [13].

In our search through the current guidelines for ART and through the latest publications of the Conference on Antiretroviral Drug Optimization, we found only one systematic review that assessed the efficacy of ART in treatment-experienced HIV-infected adults [14]. The study included all randomized clinical trials (RCT) published from 2003 to 2010 that assessed the efficacy of adding a new ART (vs placebo) to OBT for treatment-experienced HIV-infected subjects [14]. The new ARV + OBT approach vs placebo was first proposed by the TORO clinical trial [15]. Since then, this new ARV + OBT approach has been the most used rationale for evaluating new drugs for individuals with triple-class virological resistance [15–19]. However, the combinations studied have often not allowed direct comparisons between ARVs and have thus resulted in little evidence not only as to which the best combinations of two or three drugs are, but also as to which drug with a novel mechanism is to be chosen. In addition, this systematic review did not assess DTG trials, nor did it address the methodological quality and any research gaps of the RCTs included.

The objective of our systematic review and meta-analysis was twofold: (a) to assess the efficacy of third-line therapy for adults with HIV/AIDS based on RCTs that adopted the “new ARV + OBT” approach and (b) to assess the scientific evidence related to treatment strategies for multi-experienced patients under the WHO proposal of third-line therapeutic approaches.

## Methods

The PICOS criteria for inclusion are reported in Additional file 2:Table S1.

### Data sources and searches

Our systematic review comprised a search of the following electronic databases spanning January 1, 1966, to December 31, 2015: MEDLINE (accessed by PubMed), EMBASE, LILACS, ISI Web of Science, SCOPUS, and Cochrane Central Register of Controlled Trials. In addition, we searched the references of studies published in the following international scientific meetings: International AIDS Conference (2001 to 2015); Conference on Retroviruses and Opportunistic Infections (CROI) (1997 to 2015); Interscience Conference on Antimicrobial Agents and Chemotherapy (ICAAC) (2003 to 2015); and International Congress on Drug Therapy in HIV Infection (2004 to 2015).

The reason the search for publications only spanned until December 31, 2015, was because this study only aimed to assess third-line therapy in RCTs that used the OBT approach, and the last RCT known in scientific literature that used this strategy was published on March 13, 2013. The search strategy used is shown in the Additional file 4. No language restrictions were established for published studies. The present systematic review is reported in accordance with the Preferred Reporting Items for Systematic Reviews and Meta-Analyses (PRISMA) statement [20].

### Inclusion criteria

We included all RCTs that were published or presented in their complete versions. Eligible studies were those which enrolled third-line therapy patients, aged 16 or older, who received OBT plus new ARVs, or OBT plus placebo/comparison ARVs. Given that there is no standardized third-line therapy in the scientific literature or in international guidelines, we used the best definition so far to characterize treatment-experienced HIV-1-infected patients, which is patients with a documented genotypic and/or phenotypic resistance to at least one ARV of each of the three following classes: nucleoside reverse transcriptase inhibitors (NRTI), non-nucleoside reverse transcriptase inhibitors (NNRTI), and protease inhibitors (PI). New drugs included enfuvirtide (ENF), tipranavir (TPV), DRV, RAL, ETV, maraviroc (MVC), vicriviroc (VIC), amdoxovir (DAPD), and DTG.

### Exclusion criteria

The following exclusion criteria were established: studies that (a) were not randomized and/or did not have a control group for comparison, (b) did not adopt an OBT strategy for comparison, (c) did not provide efficacy and safety data, (d) assessed switch therapy and/or simplifying treatments, (e) included naïve patients, (f) included pregnant and breastfeeding women, and (g) included subjects under 16 years of age.

### Study selection

Two investigators (LPM and RSK) screened the titles and abstracts independently and revised the full text of eligible studies. Reviewers were not blinded to the authors’ identities nor to the institutions that published the manuscripts. They evaluated the full-text articles, determined study eligibility, and conducted data extraction independently, solving disagreements by consensus whenever necessary.

### Data extraction

Data from included studies was extracted using a structured data collection tool developed by the researchers, which was based on the recommendations of the Consolidated Standards of Reporting Trials (CONSORT) [21], the Cochrane Collaboration’s tool for assessing risk of bias in randomized trials [22], and the literature addressing the most important issues regarding the critical appraisal of randomized clinical trials [23–25]. The synthesized information was assessed using the Grading of Recommendations Assessment, Development and Evaluation (GRADE) [26].

### Data analysis

The data extracted from the RCTs assessed the characterization of primary and secondary outcomes, as well as efficacy, safety, subgroup analyses, and the results of said studies. The primary outcome assessed in the meta-analysis was the proportion of patients that reached undetectable HIV RNA levels (defined as < 50 copies/mL) at 48 weeks of follow-up. This outcome was based on clinical and statistical criteria and was chosen because it represents long-term cART effectivity and because most of the trials presented results for this outcome. Nonetheless, the referred definition was also an approach to reduce the heterogeneity among the studies. Trials that did not present data for the referred outcome at week 48 were considered in the analysis under another follow-up time (i.e., 16, 24, 96 weeks).

The primary outcome was also analyzed according to the number of fully active ARVs at 48 weeks of follow-up (i.e., OBT with zero, 1 or 2 + active drugs) and the risk of bias. The stratification regarding the risk of bias was implemented based on the Cochrane Collaboration’s tool for assessing risk of bias [22] and resulted in three categories: (a) high risk: 2 or more high-risk criteria or 1 criterion with high risk and 2 or more criteria with unclear risks; (b) moderate risk: 1 criterion with high risk and only one criterion with an unclear risk or no criteria with high risks and 1 or more criteria with unclear risks; and (c) low risk: all criteria with low risks.

Secondary outcomes, including any increases in CD4 + cell count and any other outcome related to a decrease in viral load, were analyzed using descriptive statistics only.

### Assessment of risk of bias

The risk assessment of the retrieved studies included random sequence generation, allocation concealment, blinding of participants and personnel, outcome assessment, incomplete outcome data, selective reporting, and other potential sources of bias [22]. Risk of bias in the RCTs was assessed using Cochrane’s Risk of Bias assessment tool [22], and the quality of the evidence available was assessed according to the GRADE [26] criteria.

### Statistical analysis

The summary measure was the risk difference between intervention and control groups considering the outcome “proportion of patients reaching undetectable HIV RNA levels (defined as < 50 copies/mL) at 48 weeks of follow-up”. Study estimates were aggregated using the random-effects model with the DerSimonian-Laird estimator and the Mantel-Haenzel method. Heterogeneity among studies was assessed through the *I*^2^ statistics and Cochran’s *Q* test. *I*^2^ values greater than 50% were considered likely to indicate substantive heterogeneity. To investigate the presence of heterogeneity among studies, an expected sub-group meta-analysis was planned according to the number of fully active drugs in the OBT (zero, 1, or 2 fully active drugs) and the study’s risk of bias (high, moderate, or low risk of bias). Risk of publication bias was assessed by a funnel plot. Analyses were performed using the Review Manager software (RevMan version 5.1) and the GRADEpro GDT software was used to synthesize the information assessed in accordance with the GRADE criteria [26].

## Results

### Study selection

**Fig. 1:**
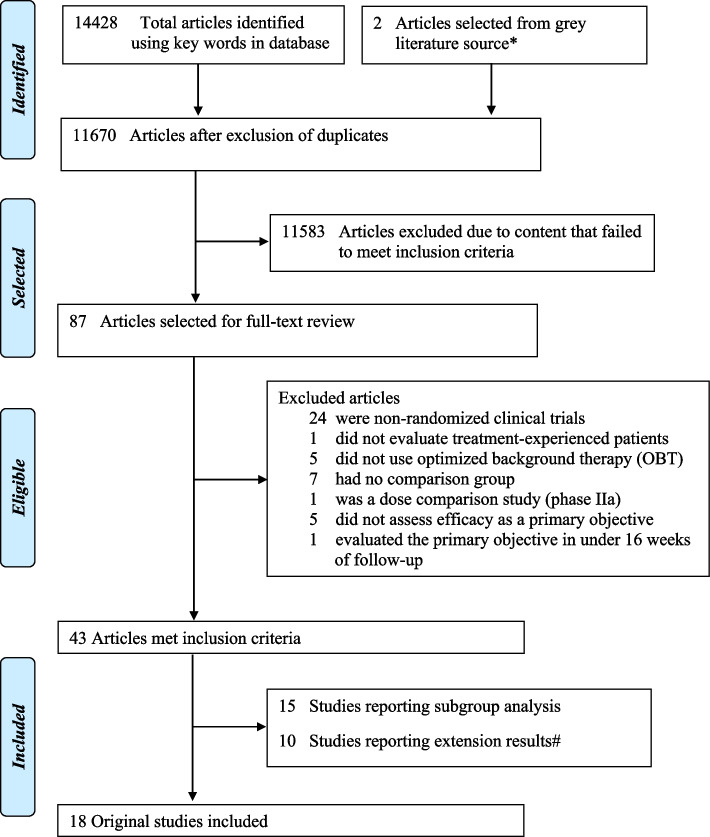
Flow chart of systematic review and study selection. *We identified 57 abstracts, including 25 whose articles were not published, 28 already identified in our peer-reviewed literature, and 4 containing exclusion criteria. Only 2 articles were selected. ^#^Some studies reporting extension results were considered in the meta-analysis instead of their original studies

Eighteen randomized controlled trials (totaling 7963 patients) comprising 47 ART comparison groups were retrieved [15–19, 27–39] (Fig. 1 and Table 1). We also identified another 10 publications reporting extension results (referring to the previously selected RCTs that presented results in more advanced follow-up periods in time, i.e., 48 and 96 weeks) and 15 studies reporting subgroup analyses (Additional file 3: Table S2). Although some studies reporting extension results were included in the meta-analysis, only the 18 original studies were considered in the final analysis that we developed.

**Table 1:** Randomized controlled trial characteristics

**Study/year (reference)**	**Drug**	N **Interv. group**	***N* Control group**	**Time**^a^** (weeks)**	**ART in Interv./control groups**	**design**	**Primary outcome**	**Results**	**NNT (CI)**	**CD4 difference (cell/μL)**
TORO-1, 2003 [15]	ENF	332	169	24	ENF + OBT/OBT	S- III	Change from baseline in plasma HIV-1 RNA level (log10 copies/mL)	64/326 vs 12/165^b^	8.1 (5.8–17.9)	44 (23–66)^d^
TORO-2, 2003 [39]	ENF	341	171	24	ENF + OBT/OBT	S- III	Change from baseline in plasma HIV-1 RNA level (log10 copies/mL)	41/335 vs 9/169^b^	14.5 (9.2–96.9)	28 (4–51)^d^
RESIST-1, 2006 [ 16]	TPV/r	313	317	24	TPV/r + OBT/PI/r + OBT	S- III	Proportion of patients with a reduction in the HIV-1 load of ≥ 1 log10	129/311 vs 69/309	5.2 (3.8–8.6)	30
RESIST-2, 2006 [28]	TPV/r	271	268	24	TPV/r + OBT/PI/r + OBT	S- III	Proportion of patients with a reduction in the HIV-1 load of ≥ 1 log10	111/271 vs 40/268	3.8 (3.0–5.4)	33
A5118, 2006 [36]	DAPD	9	9	24	DAPD + ENF + OBT/placebo + ENF + OBT	S- III	Time-averaged area under the curve minus baseline in plasma HIV-1 RNA from baseline (log10 copies/mL)	3/9 vs 1/9^c^	4.5 (2.3–∞)	19
POWER-1, 2007 [17]	DRV/r	255	63	24	DRV/r + OBT/PI + OBT	S-IIb	Proportion of patients with a reduction in the HIV-1 load of ≥ 1 log10 (TLOVR algorithm)	46/65 vs 15/63	2.1 (1.6–3.4)	104
POWER-2, 2007 [29]	DRV/r	234	60	24	DRV/r + OBT/PI + OBT	S-IIb	Proportion of patients with a reduction in the HIV-1 load of ≥ 1 log10 (TLOVR algorithm)	24/57 vs 6/53	3.2 (2.3–7.8)	47
TITAN, 2007 [37]	DRV/r	302	302	48	DRV/r + OBT/LPV/r + OBT	NI-III	Proportion of patients achieving viral load < 400 copies/mL	228/298 vs 199/297	10.5 (5.9–51.5)	7
DUET-1, 2007 [30]	ETR	304	308	24	ETR + OBT/placebo + OBT	S- III	Proportion of patients achieving viral load < 50 copies/mL (TLOVR algorithm)	170/304 vs 119/308	5.8 (3.9–11.0)	25
DUET-2, 2007 [31]	ETR	295	296	24	ETR + OBT/placebo + OBT	S- III	Proportion of patients achieving viral load < 50 copies/mL (TLOVR algorithm)	183/295 vs 129/296	5.4 (3.8–9.9)	12
TMC125-C223, 2007 [32]	ETR	79	40	24	ETR + OBT/OBT	S-IIb	Change from baseline in plasma HIV-1 RNA level (log10 copies/mL)	14/79 vs 3/40^b^	9.8 (5.4–∞)	38
Grinsztejn, 2007 [38]	RAL	45	45	24	RAL + OBT/placebo + OBT	S-IIb	Change from baseline in plasma HIV-1 RNA level (log10 copies/mL)	25/45 vs 6/45^b^	2.4 (1.7–4.7)	113 (76–150)^d^
BENCHMRK 1e2, 2008 [18]	RAL	466	237	16	RAL + OBT/placebo + OBT	S- III	Proportion of patients achieving viral load < 400 copies/mL	355/458 vs 99/236	2.8 (2.3–3.6)	64
MOTIVATE 1e2, 2008 [19]	MVC	434	214	48	MVC + OBT/placebo + OBT	S-IIb	Change from baseline in plasma HIV-1 RNA level (log10 copies/mL)	194/426 vs 35/209^b^	3.5 (2.8–4.7)	63 (44–82)^d^
A4001029, 2009 [33]	MVC	63	64	24	MVC + OBT/OBT	S-IIb	Change from baseline in plasma HIV-1 RNA level (log10 copies/mL)	14/52 vs 9/58^b^	8.8 (3.8–∞)	27 (1–52)^d^
VICTOR-E1, 2010 [34]	VIC	39	37	48	VIC + OBT/placebo + OBT	S-IIb	Change from baseline in plasma HIV-1 RNA level (log10 copies/mL)	22/39 vs 5/35^b^	2.4 (1.7–5.5)	39
VICTOR-E3 and E4, 2012 [ 21]	VIC	571	286	48	VIC + OBT/placebo + OBT	S-III	Proportion of patients achieving viral load < 50 copies/mL	313/486 vs 145/235	37 (− 21 to 10)	11
SAILING, 2013 [ 29]	DOL	360	364	48	DOL + OBT/RAL + OBT	NI-III	Proportion of patients achieving viral load < 50 copies/mL	251/354 vs 230/361	13.9 (7.1–296.6)	9

*Interv.* intervention, *ENF* enfuvirtide, *TPV* tipranavir, *r* ritonavir, *DAPD* amdoxovir, *DRV* darunavir, *LPV* lopinavir, *ETR* etravirine, *RAL* raltegravir, *MVC* maraviroc, *VIC* vicriviroc, *DOL* dolutegravir, *PI* protease inhibitor, *NNT* number needed to treat, *CI* confidence interval, *S* superiority, *NI* non-inferiority, *III* phase III study, *IIb* phase IIb study, *OBT* optimized background therapy, *TLOVR* time to loss of virologic response.

^a^Time to which the study was designed a priori

^b^Results and NNT presented from the secondary outcome “proportion of patients achieving viral load < 50 copies/mL.”

^c^Results and NNT presented from the secondary outcome “proportion of patients achieving viral load < 200 copies/mL.”

^d^Difference confidence interval. In phase IIb studies, the author’s recommended dose for experimental group or dose used in further studies was selected

### Study characteristics

The 18 RCTs assessed the efficacy and safety of nine new ARVs:ENF [15, 39], TPV [16, 28], DAPD [36], DRV [17, 29, 37], ETV [30–32], RAL [18, 38], MVC [19, 33], VIC [27, 34], and DTG [35] (Table 1). Eleven of those studies (61%) were characterized as phase III trials and seven as phase IIb studies. No post-commercialization study was retrieved. Length of follow-up varied among the studies: one study lasted 16 weeks [18], twelve studies lasted 24 weeks [15–17, 28–33, 36, 38, 39], and five studies assessed patients after 48 weeks of follow-up [19, 27, 34, 35, 37]. All but two studies were superiority trials [35, 37]. A substantial variation was observed regarding the definition of the primary outcomes of the studies.

### Treatment efficacy

**Fig. 2:**
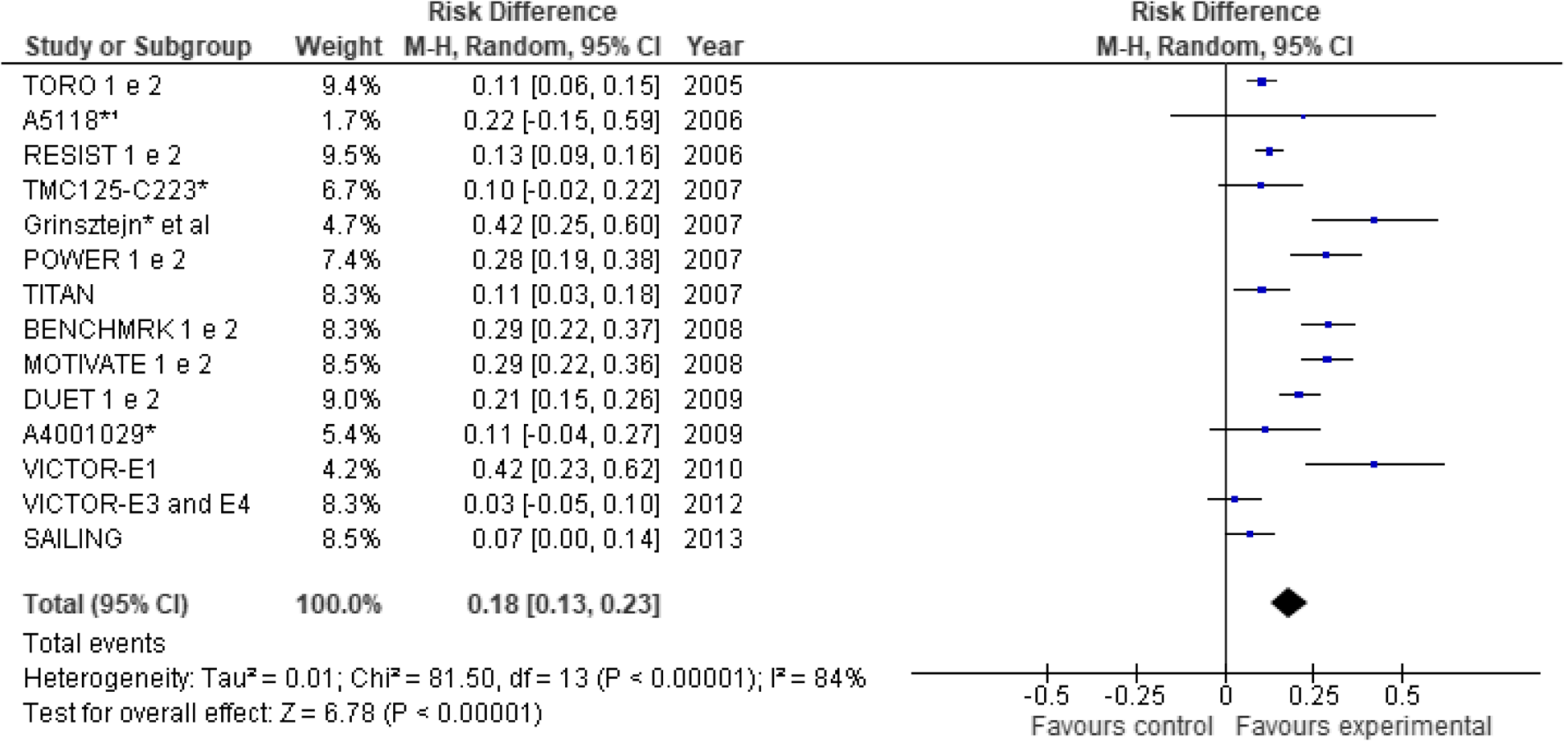
Meta-analysis comparing treatment and control groups. *Studies presenting data at week 24. Outcome: proportion of patients with < 200 HIV-1 RNA copies/mL. Outcome: proportion of patients with < 50 HIV-1 RNA copies/mL at week 48. Obs. BENCHMRK studies present data in a 96-week follow-up

It is important to note that some trials reporting extension results were also considered in our meta-analysis—as long as their findings were of 48 weeks [40–43]. Therefore, it is worth clarifying that the number of studies analyzed reduced from 18 to 14 because some publications aggregated the results of two original studies into a single trial reporting extension results [40–43]. Efficacy results are shown based on the proportion of patients that achieved HIV RNA viral load results below 50 copies/ml at 48 weeks of follow-up (Fig. 2). Of the 14 trials [18, 19, 27, 32–38, 40–43], nine trials demonstrated the superiority of the new ARVs plus the studied combination in comparison to OBT control groups, thus demonstrating the efficacy of said ARVs [18, 19, 34, 37, 38, 40–43]. The other 5 trials did not demonstrate the efficacy of the new ARVs + OBT in comparison to the control group [27, 32, 33, 35, 36]. The pooled measure showed that individuals who received the new investigational drug containing an ARV combination were more likely (18%) to achieve viral undetectability in comparison to control groups: risk difference was 0.18 (95% CI 0.13–0.23) (*I*^2^ = 84%, *P* < 0.00001).

**Fig. 3:**
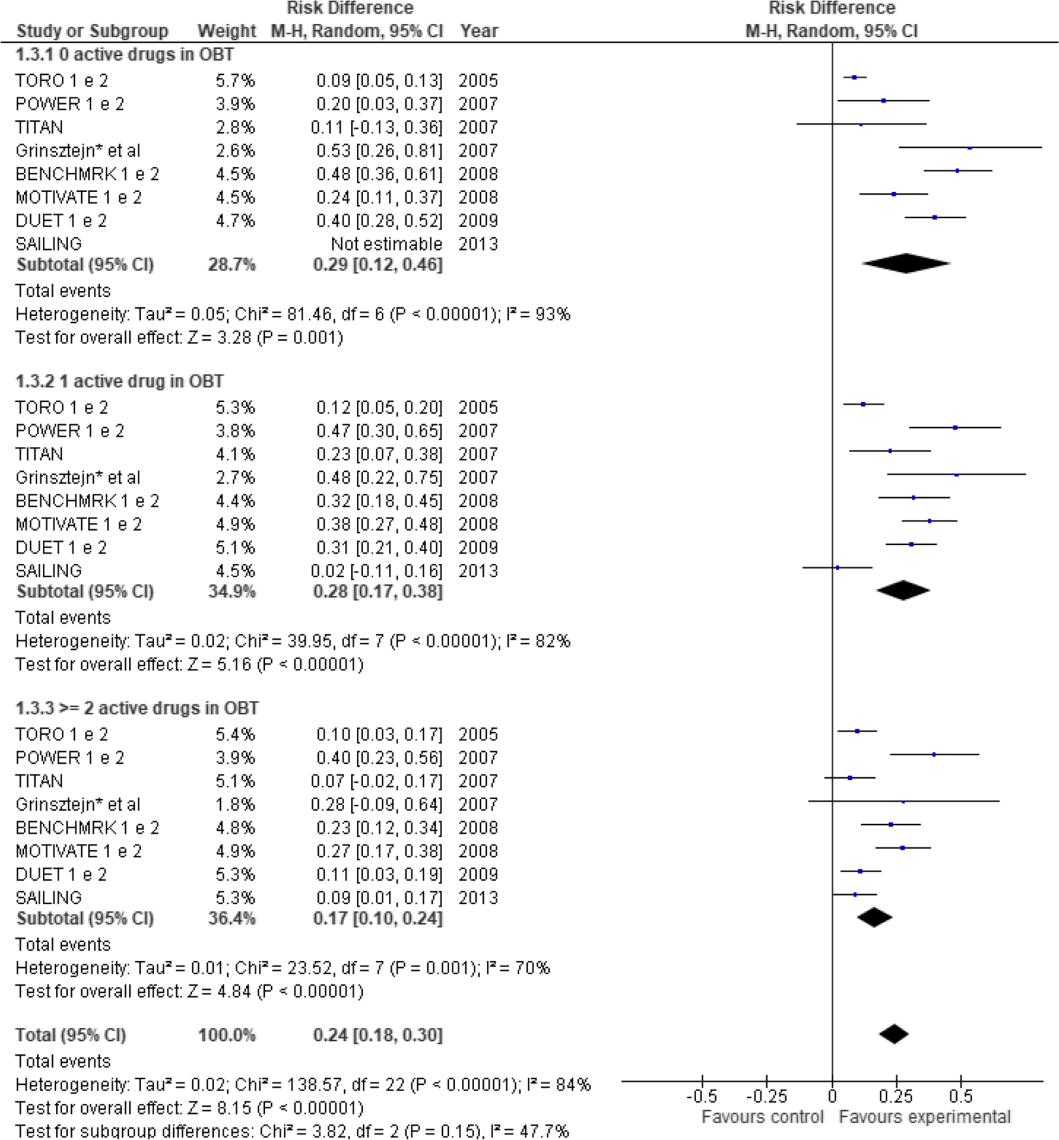
Meta-analysis comparing treatment and control groups stratified by the number of active drugs in OBT. RD, risk difference; CI, confidence interval. *Studies presenting data at week 24. ^1^Outcome: proportion of patients with < 200 HIV-1 RNA copies/mL. Outcome: proportion of patients with < 50 HIV-1 RNA copies/mL at week 48. Obs.1: BENCHMRK studies present data in a 96-week follow-up. Obs.2: SAILING study presented results stratified by active drugs in OBT only in 2 categories (< 2 and ≥ 2 active drugs in OBT). Data of category < 2 active drugs in OBT were presented in subgroup “1 active drug in OBT”

When analyzing the outcome “proportion of patients that achieved HIV RNA viral load results below 50 copies/ml at 48 weeks of follow-up” stratified by the number of fully active ARVs (OBT with zero, 1 or 2 + active drugs), only 8 studies were considered [18, 19, 35, 37, 38, 40, 42, 43], because these were the only ones that provided data results according to such stratification (Fig. 3). Some studies only presented these results in the extension follow-up [44] or in the subgroup analysis [45]. The SAILING study [35] presented results stratified by active drugs in OBT in 2 categories only (< 2 and ≥ 2 active drugs in OBT). Therefore, data of category < 2 active drugs in OBT were presented in the subgroup “1 active drug in OBT,” and data was not estimable in the subgroup “0 active drugs in OBT.” Risk difference among strata with zero, one, and two or more fully active drugs was 0.29 (95% CI 0.12–0.46), 0.28 (95% CI 0.17–0.38), and 0.17 (95% CI 0.10–0.24), respectively. That means that the difference between the proportion of patients who reached an RNA viral load below 50 copies/ml decreased from zero/one to two/more fully active drugs, yet this difference was not statistically significant. The pooled risk difference considering the three strata (i.e., OBT with zero, 1, and 2 or more active drugs) was 0.24 (95% CI 0.18–0.30).

**Fig. 4:**
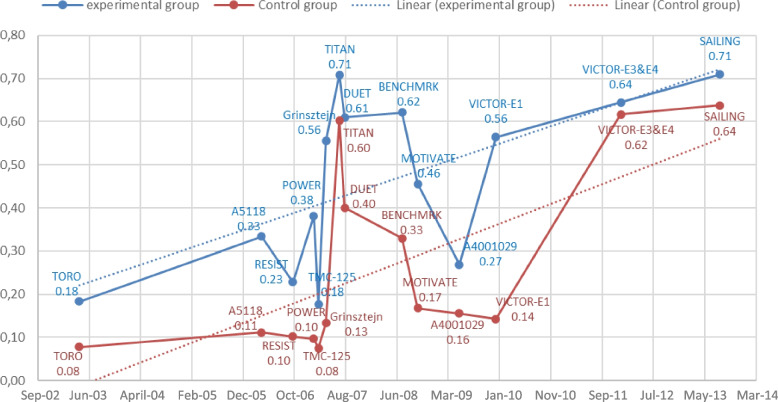
Time evaluation of the proportion of patients with < 50 HIV-1 RNA copies/mL at week 48

Over the period analyzed, we observed a linear rising tendency in viral suppression rates in both trial and control groups, starting from the TORO and RESIST trials [40, 41], with respectively 18% vs 8% and 23% vs 10%, to the SAILING trial [35], with rates of 71% vs 64% (Fig. 4). However, in this same outcome, we identified a smaller difference over the entire period (space between the dotted lines) when comparing the trial and control groups.

Two studies [16, 28] adopted genotypic sensitivity scores, six used phenotypic scores [15, 30, 31, 37–39], and five studies used both methods, which resulted in an overall sensitivity score [18, 19, 27, 33, 35]. The remaining studies did not provide data on the method chosen for the detection of viral resistance [17, 29, 32, 34, 36].

All RCTs assessed CD4 cell count as a secondary outcome. The maximum and minimum increase in CD4 cell count when intervention groups were compared with control groups at week 24 were 19 and 108 cells/mm3, respectively [15–17, 28–33, 36, 38, 39], and 7 and 67 cells/mm3 respectively at week 48 [19, 27, 34, 35, 37]. The increase in CD4 cell count at week 16 was 64 cells/mm3 [18] (Table 1). The average increase in CD4 cell count was not calculated due to the large heterogeneity in the follow-up time of the studies that presented such data.

Furthermore, only seven studies analyzed disease progression outcomes [16, 18, 28, 30, 31, 35, 37]. Fourteen trials presented results related to mortality [16–19, 27–35, 37].

### Risk of bias

**Fig. 5:**
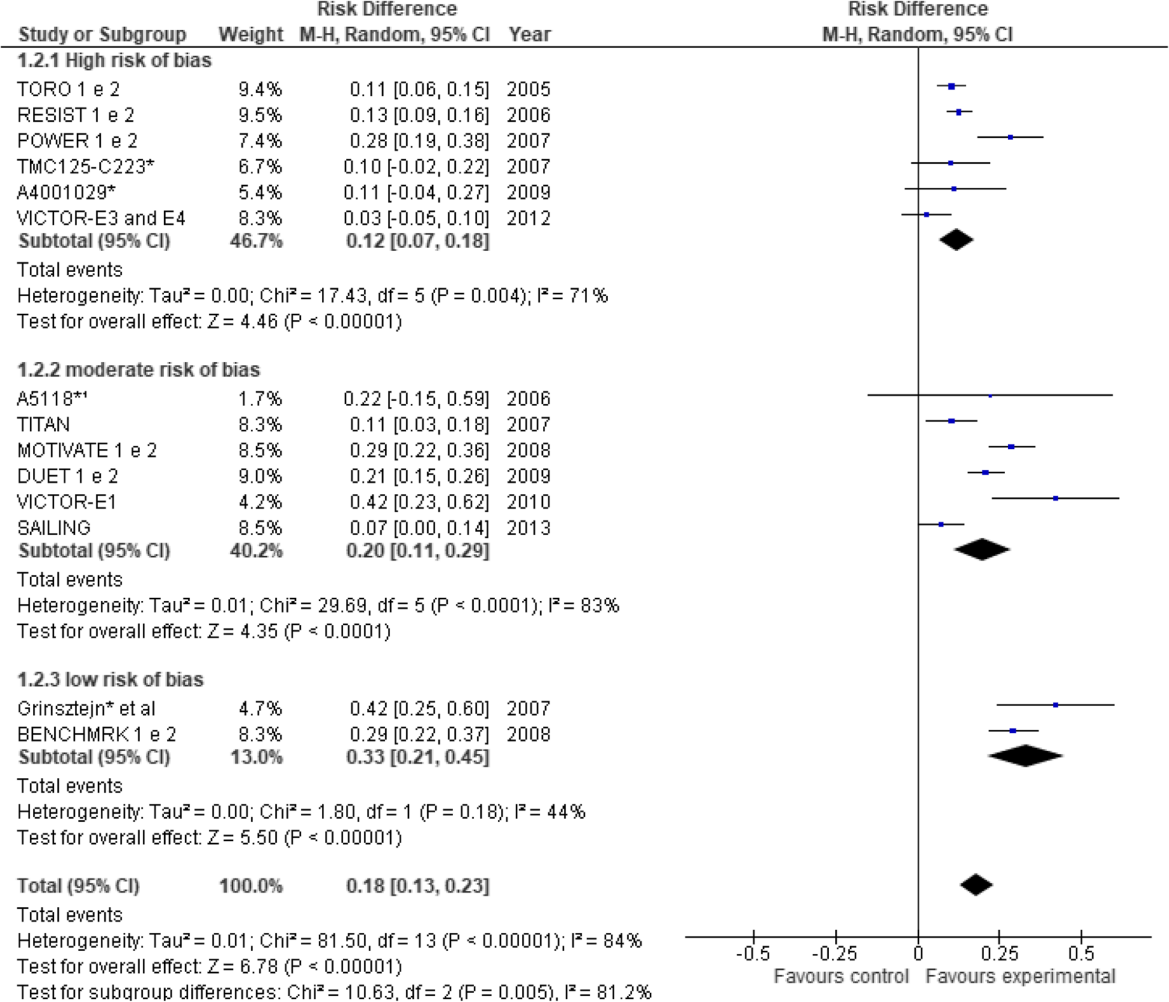
Meta-analysis comparing treatment and control groups stratified by study risk of bias. RD, risk difference; CI, confidence interval. *Studies presenting data at week 24. ^1^Outcome: proportion of patients with < 200 HIV-1 RNA copies/mL. Outcome: proportion of patients with < 50 HIV-1 RNA copies/mL at week 48. Risk of bias categories: *high risk of bias* (high risk of bias in 2 or more criteria, according to Cochrane Collaboration’s tool for assessing risk of bias, or high risk of bias in 1 criterion and unclear risk of bias in 2 or more criteria), *moderate risk of bias* (high risk of bias in only 1 criterion and unclear risk of bias in only 1 criterion, or no criteria with high risk of bias and unclear risk of bias in 1 or more criteria), and *low risk of bias* (all criteria with low risk of bias). Obs. BENCHMRK studies present data in a 96-week follow-up

The studies were analyzed in relation to the outcome “proportion of patients with < 50 HIV-1 RNA copies/mL at week 48” stratified by study risk of bias according to the Cochrane Collaboration’s tool [22]. The results presented three categories: (a) high risk [15–17, 27–29, 32, 33, 39]; (b) moderate risk [19, 30, 31, 34–37]; and (c) low risk [18, 38] (Fig. 5). Pooled risk difference between intervention and control groups varied significantly (*p*-value < 0.001 in the overall test) among subgroups according to the risk of bias, with 0.12 (95% CI 0.07–0.18), 0.20 (95% CI 0.11–0.29), and 0.33 (95% CI 0.21–0.45) for high, moderate, and low risk of bias respectively. Pairwise comparisons using the Wald test showed a significant difference between high and low risk of bias subgroups (*p*-value = 0.0045). Although not significant for the high vs. moderate risk (*p*-value = 0.1602) and for the moderate vs. low risk (*p*-value = 0.0630), a tendency was observed among the subgroups, showing that the lower the risk of bias in the studies, the greater the risk difference between intervention and control group.

**Fig. 6:**
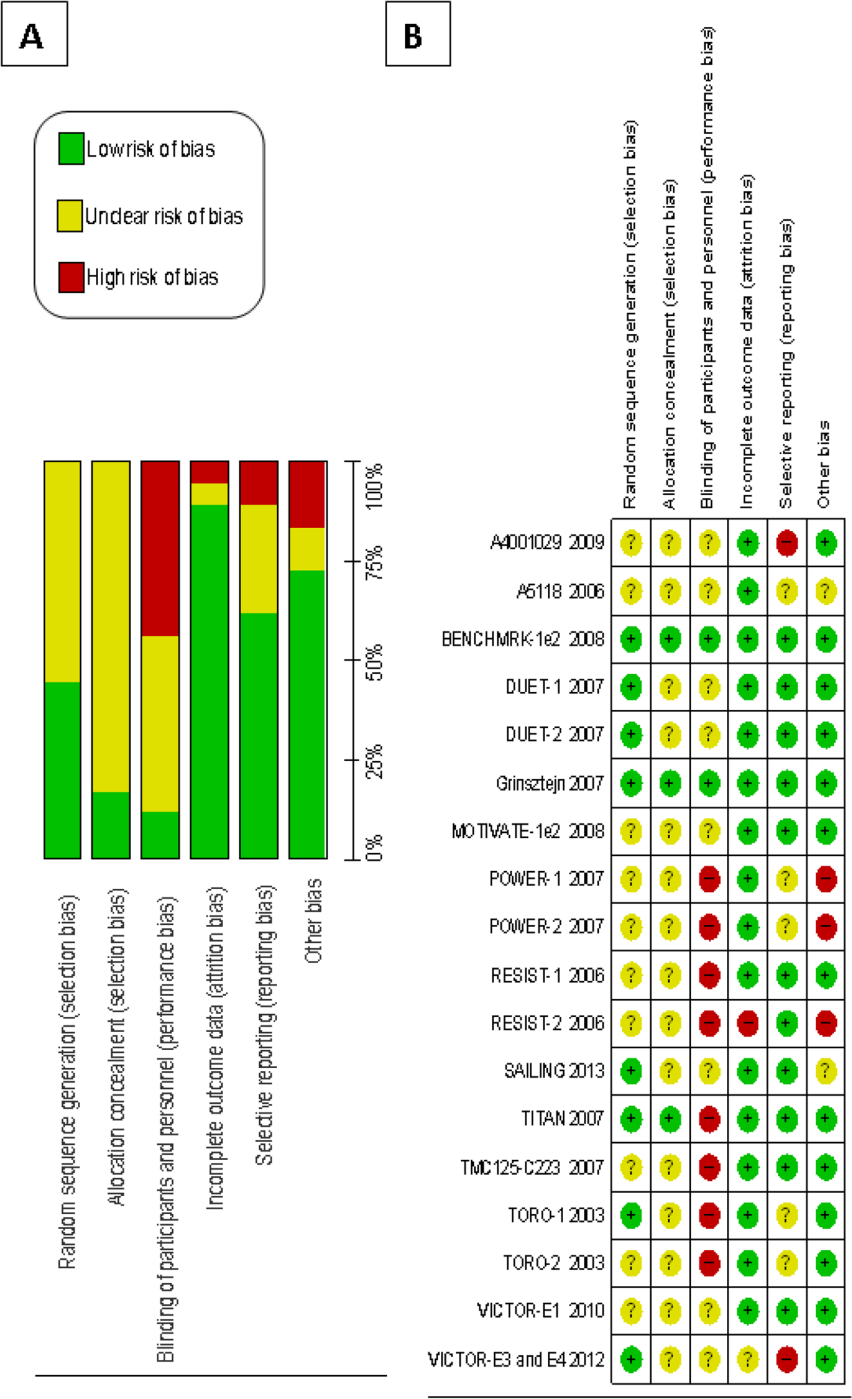
Risk of bias graph and summary of the 18 RCT analyzed. **A** Risk of bias graph. Review authors’ judgements about each risk of bias item presented as percentages across all included studies. **B** Risk of bias summary. Review authors’ judgements about each risk of bias item for each included study

Ten out of the 18 studies (55.5%) did not provide enough information for us to assess the method used to generate the sequence of randomization [16, 17, 19, 28, 29, 32–34, 36, 39] (Fig. 6A, B). Fifteen studies (83.3%) did not clarify the procedures adopted for allocation concealment [15–17, 19, 27–36, 39]. High risk of performance bias due to lack of participant and researcher blinding was found in eight (44.4%) studies [15–17, 28, 29, 32, 37, 39].

### Risk of publication assessment

Despite the relatively small number of retrieved RCTs, the funnel plot (Additional file 1: Figure) does not suggest publication bias.

### Quality of evidence

We assessed the recommendation levels and quality of evidence findings according to the GRADE criteria [26] (Table 2). We observed that the subjects who received new investigational drugs were more likely (47.8%) to achieve the outcome (i.e., less than 50 copies/ml) when compared to control subjects (RR 1.5; 95% CI 1.4–1.6), thus resulting in moderate quality of evidence.

**Table 2:** GRADE summary of findings

New ARV + OBT compared to placebo or standard ARV + OBT for HIV-1-infected patients with resistance to 3 classes of ARV					
**Patient or population:** patients with HIV-1 infected patients with resistance to 3 classes of ARV **Settings:** **Intervention:** new ARV + OBT **Comparison:** placebo or standard ARV + OBT					
**Outcomes**	**Illustrative comparative risks**^a^** (95% CI)**		**Relative effect** **(95% CI)**	**No. of Participants** **(studies)**	**Quality of the evidence** **(GRADE)**
	Assumed risk	Corresponding risk			
	**Placebo or standard ARV + OBT**	**New ARV + OBT**			
**proportion of patients achieving viral load < 50 copies/ml** Follow-up: mean 48 weeks	**315 per 1000**	**465 per 1000** (438 to 493)	**RR 1.478** (1.392 to 1.568)	7709 (18 studies)	⊕ ⊕ ⊕ ⊝ **moderate**^b,c,d^
**CD4 cell count increase** Follow-up: mean 48 weeks	The mean cd4 cell count increase in the control groups was **57.21 mean change in CD4 count from baseline (cells/ul)**	The mean cd4 cell count increase in the intervention groups was **40.22 higher** (38.54 to 41.89 higher)		7689 (18 studies)	⊕ ⊝ ⊝ ⊝ **very low**^b,c,e^
GRADE Working Group grades of evidence **High quality:** Further research is very unlikely to change our confidence in the estimate of effect **Moderate quality:** Further research is likely to have an important impact on our confidence in the estimate of effect and may change the estimate **Low quality:** Further research is very likely to have an important impact on our confidence in the estimate of effect and is likely to change the estimate **Very low quality:** We are very uncertain about the estimate					

*CI* confidence interval, *RR* risk ratio

^a^The basis for the assumed risk (e.g., the median control group risk across studies) is provided in footnotes. The corresponding risk (and its 95% confidence interval) is based on the assumed risk in the comparison group and the relative effect of the intervention (and its 95% CI)

^b^Most studies have more than one item with high risk of bias or uncertain risk of bias. Only 2 of 16 studies evaluated have low risk of bias in all 6 criteria

^c^The studies evaluated different ARV and the comparison group varied between studies. The OBT design does not allow direct assessment of treatment effectiveness at an individual level

^d^Most studies present favorable results to experimental group

^e^The difference in CD4 increase between the experimental and control groups varied widely from study to study

New investigational ARV groups were associated with an average increase of 40.2 cells/mm^3^ in CD4 count in comparison to the control group. The evaluation of evidence quality was rated as very low. Consequently, the corresponding effect estimates of those findings are very imprecise.

## Discussion

The efficacy results demonstrated that the groups that received the new ARV + OBT were more likely to achieve viral suppression when compared to the control groups. Nine trials established such superiority [18, 19, 34, 37, 38, 40–43] and the pooled measure confirms this finding (risk difference 0.18, 95% CI 0.13–0.23) (Fig. 2). Individuals who received the ARV combination with the new drugs were 18% more likely to achieve viral undetectability when compared to control groups. In addition, the studies showed that new drugs provide CD4 cell count recovery, even though the magnitude of this increase was notably modest (mean of 40 cell/μL).

All categories demonstrated statistical significance in favor of the experimental group when considering the achievement of an HIV RNA viral load below 50 copies/ml at 48 weeks of follow-up according to the number of active drugs in the OBT regimen (i.e., zero, one, and two or more fully active drugs). However, although we did find some risk differences, our meta-analysis did not reach statistical significance among the different strata. Even so, according to the tendency observed in Fig. 3, we observed that adding a novel drug to the OBT might have slightly less effect in achieving complete viremia suppression when there are two or more active drugs in the OBT. To the best of our knowledge, this is the first meta-analysis that has made such a comparison. Besides, our results support the evidence that the greater the number of active drugs in the therapeutic regimen, the higher the chance of viral suppression, no matter which drugs are used in the OBT. Pichenot et al.’s meta-analysis, which assessed the efficacy of ART in treatment-experienced HIV-infected adults, demonstrated that the most important predictive factor for achieving undetectable HIV RNA was the number of fully active drugs included in the regimen [14], which is in agreement with our findings.

Among the 18 RCTs included in our study, only two [18, 38] showed low risk of bias according to the 6 methodological evaluation criteria used [22], a finding which therefore demonstrates that most of the studies were prone to bias (Fig. 6B). In addition, we observed that the lower the risk of bias in the studies, the greater the risk difference between the comparison groups (Fig. 5), thus emphasizing the importance of assessing the risk of bias in studies, as well as its influence on the efficacy results.

A study that conducted a bibliometric analysis of 103 HIV reviews found that further HIV trials are necessary and that it is essential for future trials to incorporate strategies that reduce the risk of bias, since design and methodological flaws have limited the usability of the findings [46]. According to the GRADE criteria, further research is needed to achieve more stable evidence related to efficacy outcomes (Table 2). Besides, even the recommendations of specific drugs [6–8] were considered a 1C grading of evidence, characterized as potentially biased, thus stemming from trials with serious flaws and with uncertain effect estimates.

One of the objectives of this systematic review was to analyze the scope of existing evidence on third-line therapy provided by the scientific literature, so as to produce data to support guidelines that would recommend the best antiretroviral schemes that patients should take through a staggered approach. However, third-line ART still lacks an operational definition. Some recent publications that used the term “third-line therapy” were based on drugs with a high genetic barrier to resistance [47–49]. One such publication is an observational study developed in Southern Africa [48] while the others are retrospective studies carried out in Johannesburg [47] and in Latin America [49], and all of which defined third-line regimens as being those that use newer generation NNRTIs like etravirine and darunavir, as well as the integrase inhibitor raltegravir. Consequently, due to this lack of a single clear definition, there was a high heterogeneity related to the third-line drug scheme chosen for these studies.

Nevertheless, the comparisons made by the trials that we included do not allow for recommendations as to which drugs the multi-experienced patients should use, given that the 18 studies were essentially designed for regulatory purposes. Consequently, the evidence summarized herein is not yet enough to answer which antiretroviral combinations are more effective for therapeutic regimens that are used sequentially—even more so considering the individualized needs of patients with documented multi-drug resistance. This does not mean that future studies will not be able to demonstrate such achievement, but rather that the RCTs analyzed did not assess which antiretroviral combinations are the most effective. Furthermore, a review developed by Vitoria and coworkers concluded that sequencing first-line, second-line, and third-line regimens will allow better planning by providing a rationale for the choice and the number of regimens that programs need to obtain [50].

Therefore, it is our understanding that further studies should be carried out aiming specifically to define the best ART combinations to be used for HIV-experienced patients who require third-line therapy. Such findings would provide essential information to improve procurement and logistics, all the while providing much needed evidence-based consistency of treatment that would reduce the uncertainty that is experienced at this stage of treatment.

This systematic review has some limitations. It presents only efficacy data, as the analysis of the safety profile of the antiretroviral combinations described here did not present uniform definitions of adverse events, in addition to the fact that most clinical trials are of the phase II and phase III type, therefore, not including a more detailed assessment of the safety of the evaluated drugs. Furthermore, it must be stated that the studies evaluating new drugs in experienced patients are essentially based on surrogate outcomes. We did not assess outcomes related to viral resistance. Moreover, these trials were developed mostly in high- and middle-to-high-income countries. Most subgroup analyses evaluating multidrug-resistance profiles in the existing studies presented post hoc analyses and a small number of patients. Presumably, the substantial heterogeneity found relies potentially on the differences in the assessments of the primary outcomes within the studies, as well as the aforementioned differences in the length of the follow-up study period. A high degree of heterogeneity between studies still remained even though we used strategies to reduce it, such as defining a primary outcome that is widely used, as well as a defined follow-up time and the development of effectiveness analyses according to the number of fully active drugs in the OBT. This happened due to the large number of cARTs analyzed, a substantial variation in the definition of the resistance criteria for ART, differences in the assessment of such resistance among the different existing phenotypic and genotypic tests [23], the risk of bias categories that each trial belongs to, and the distinct approaches to manage effectiveness data and study design.

When compared to Pichenot et al.’s meta-analysis [14], the present study innovated in assessing the virological success rate according to the number of fully active drugs in OBT and added more relevant studies, such as A5118 [36], TITAN [37], Grinsztejn et al. [38], and SAILING [35]. Also, unlike the 2012 publication [14], our study assessed the risk of bias using Cochrane’s Risk of Bias assessment tool and analyzed the recommendation levels together with the quality of evidence according to the GRADE criteria [26].

More than 15 years have elapsed since the first RCT adopting an OBT approach for experienced HIV-infected subjects was published [15, 39]. However, despite the new ARV benefits shown in the studies in the “OBT-era,” the future of OBT-based RCTs evaluating new drugs is controversial due to the growing difficulty to establish superiority in a context of therapeutic combinations that have become progressively more powerful [51]. Figure 3 shows an increasing tendency during the investigated period for viral suppression rates to be higher in recent studies than in older ones. This rising trend emerged in both comparison groups, experimental and control, and the differences between them have been decreasing over time. Such a result is in accordance with the evidence referred to above, indicating a scenario of limited use of OBT strategies to demonstrate the efficacy of new ARVs. There are, however, two recent studies that have used the OBT approach: the first, a clinical trial developed across 23 countries that evaluated the efficacy of fostemsavir in adults with multidrug-resistant HIV-1 infection [52], and the second, a retrospective analysis using secondary data to assess the efficacy of dolutegravir in antiretroviral-experienced patients over a 5-year follow-up period [53].

Novel trial designs for new antiretroviral drugs intended for use with treatment-experienced HIV-infected patients on a failing regimen have been suggested in the past [54], yet their benefits remain to be better assessed, especially regarding efficacy and safety data. Despite unequivocal advances related to therapeutic options for experienced patients, the findings shown here suggest that such evidence has not been fully assessed over the years. Though studies with treatment-experienced HIV-infected patients are necessary and must be developed, they should start from an operational definition of what third-line therapy is in effect. Moreover, these trials must be performed in low- and middle-income countries and must evaluate outcomes of disease progression and mortality. After regulatory goals have been accomplished, explanatory RCTs could be replaced by pragmatic RCTs [55] that are capable of effectively assessing which antiretroviral therapy combinations for experienced-patients allow for clinically relevant results to be achieved.

## Conclusion

Our findings demonstrated that the groups of multi-experienced patients that received the new ARV + OBT presented a better chance of achieving viral suppression in comparison to control groups, even when the analysis was stratified according to the number of active drugs in the OBT regimen. Nevertheless, we found some risk difference among strata and a tendency supporting the evidence that the greater the number of active drugs in the therapeutic regimen, the higher the chance of viral suppression, no matter what drugs are used in the OBT. Furthermore, among the eighteen RCTs analyzed, only two showed low risk of bias, a finding which demonstrates how prone to bias the studies might be. As to the scope of evidence on third-line ART, we found that third-line schemes are highly heterogeneous.

Finally, once again, it is important to point out that the RCTs included in this study were essentially designed for regulatory purposes, thus resulting in insufficient evidence to define which combinations are the most effective, especially in a public health approach through clinical recommendations for third-line regimens. New studies with a clear operational definition of third-line therapy and using a sequential cART approach for treatment-experienced patients should be developed in order to enable the creation of better guidelines/schemes including the evaluation of their efficacy and safety.

## Supplementary Information

**Additional file 1. Figure.** Funnel plot of included articles in thisstudy (squares).

**Additional file 2. Table S1.** PICOS table summarizingstudy rationale.

**Additional file 3. Table S2.** Studies that reported extension results or subgroup analyses.

**Additional file 4. **Search strategy used.

## Data Availability

All data generated or analyzed during this study are included in this published article and its supplementary information files: [15–19, 27–43].

## Abbreviations

AIDS: Acquired immune deficiency syndrome
ART: Antiretroviral therapy
cART: Combined antiretroviral therapy
ARV: Antiretroviral
CONSORT: Consolidated Standards of Reporting Trials
CROI: Conference on Retroviruses and Opportunistic Infections
DAPD: Amdoxovir
DRV: Darunavir
DRV/r: Darunavir/ritonavir
DTG: Dolutegravir
ENF: Enfuvirtide
ETV: Etravirine
GRADE: Grading of Recommendations Assessment, Development and Evaluation
HAART: Highly active antiretroviral therapy
ICAAC: Interscience Conference on Antimicrobial Agents and Chemotherapy
MVC: Maraviroc
NNRTI: Non-nucleoside reverse transcriptase inhibitor
NRTI: Nucleoside reverse transcriptase inhibitor
OBT: Optimized background therapy
PI: Protease inhibitor
PRISMA: Preferred Reporting Items for Systematic Reviews and Meta-Analyses
RAL: Raltegravir
RCT: Randomized clinical trial
RevMan: Review Manager
TPV: Tipranavir
TPV/r: Tipranavir/ritonavir
UNAIDS: Joint United Nations Programme on HIV/AIDS
VIC: Vicriviroc
WHO: World Health Organization
