# Supplementary figures and images for: A systematic review and meta-analysis assessing antiretroviral therapy for treatment-experienced HIV adult patients using an optimized background therapy approach: is there evidence enough for a standardized third-line strategy?
Source: Syst Rev. 2022 Nov 17;11:243. doi: 10.1186/s13643-022-02102-3 (PMC9673282; doi:10.1186/s13643-022-02102-3)

**APPENDIX** – **Figure**


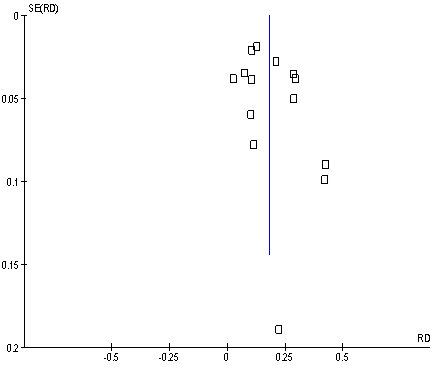


**Appendix Figure** – Funnel plot of included articles in this study (squares)

Supplement: Supplementary file 1 — Additional file 1. Figure. Funnel plot of included articles in thisstudy (squares). [file 13643_2022_2102_MOESM1_ESM.docx]
