# Supplementary material for: A systematic review and meta-analysis assessing antiretroviral therapy for treatment-experienced HIV adult patients using an optimized background therapy approach: is there evidence enough for a standardized third-line strategy?
Source: Syst Rev. 2022 Nov 17;11:243. doi: 10.1186/s13643-022-02102-3 (PMC9673282; doi:10.1186/s13643-022-02102-3)
Supplement: Supplementary file 2 — Additional file 2. Table S1. PICOS table summarizingstudy rationale. [file 13643_2022_2102_MOESM2_ESM.docx]

**APPENDIX Table 1 – PICOS table summarizing study rationale**

| Participants | Treatment-experienced HIV-1-infected subjects with 16 years of age or more |
| --- | --- |
| Interventions | New ARVs plus OBT scheme |
| Comparisons | Placebo/comparison ARVs plus OBT scheme |
| Outcomes | Proportion of patients reaching undetectable HIV RNA levels at 48 weeks of follow-up |
| Study design | Systematic review and meta-analysis |
