# Supplementary material for: A systematic review and meta-analysis assessing antiretroviral therapy for treatment-experienced HIV adult patients using an optimized background therapy approach: is there evidence enough for a standardized third-line strategy?
Source: Syst Rev. 2022 Nov 17;11:243. doi: 10.1186/s13643-022-02102-3 (PMC9673282; doi:10.1186/s13643-022-02102-3)
Supplement: Supplementary file 3 — Additional file 3. Table S2. Studies that reported extension results or subgroup analyses. [file 13643_2022_2102_MOESM3_ESM.docx]

| **APPENDIX Table 2 – Studies that reported extension results or** **subgroup analyses** | | | | |
| --- | --- | --- | --- | --- |
| **Title** | **Authors** | **Year** | **Drug** | **Journal** |
| **Studies presenting extension results** | | | | |
| Durable efficacy of enfuvirtide over 48 weeks in heavily treatment-experienced HIV-1-infected patients in the T-20 versus optimized background regimen only 1 and 2 clinical trials. | Nelson M, Arastéh K, Clotet B, Cooper DA, Henry K, Katlama C, et al. | 2005 | ENF | J Acquir Immune Defic Syndr |
| Durable efficacy of tipranavir-ritonavir in combination with an optimised background regimen of antiretroviral drugs for treatment-experienced HIV-1-infected patients at 48 weeks in the Randomized Evaluation of Strategic Intervention in multi-drug resistant patients with Tipranavir (RESIST) studies: an analysis of combined data from two randomised open-label trials. | Hicks CB, Cahn P, Cooper DA, Walmsley SL, Katlama C, Clotet B, et al. | 2006 | TPV/r | Lancet |
| TORO: ninety-six-week virologic and immunologic response and safety evaluation of enfuvirtide with an optimized background of antiretrovirals. | Reynes J, Arastéh K, Clotet B, Cohen C, Cooper D a, Delfraissy J-F, et al. | 2007 | ENF | AIDS Patient Care STDS |
| Efficacy and safety of darunavir-ritonavir at week 48 in treatment-experienced patients with HIV-1 infection in POWER 1 and 2: a pooled subgroup analysis of data from two randomised trials. | Clotet B, Bellos N, Molina JM, Cooper D, Goffard JC, Lazzarin A, et al. | 2007 | DRV/r | Lancet |
| Efficacy and safety of etravirine (TMC125) in treatment-experienced HIV-1-infected patients: 48-week results of a phase IIb trial. | Cohen CJ, Berger DS, Blick G, Grossman HA, Jayaweera DT, Shalit P, et al. | 2009 | ETR | AIDS |
| Efficacy and safety of etravirine in treatment-experienced HIV-1 patients: pooled 48 week analysis of two randomized, controlled trials. | Katlama C, Haubrich R, Lalezari J, Madruga V, Molina J, Lazzarin A, et al. | 2009 | ETR | AIDS |
| Efficacy and safety of etravirine at week 96 in treatment-experienced HIV type-1-infected patients in the DUET-1 and DUET-2 trials. | Katlama C, Clotet B, Mills A, Trottier B, Molina JM, Grinsztejn B, et al. | 2010 | ETR | Antivir Ther |
| Long-term efficacy and safety of Raltegravir combined with optimized background therapy in treatment-experienced patients with drug-resistant HIV infection: week 96 results of the BENCHMRK 1 and 2 Phase III trials. | Steigbigel RT, Cooper D a, Teppler H, Eron JJ, Gatell JM, Kumar PN, et al. | 2010 | ETR | Clin Infect Dis |
| Week 96 efficacy, virology and safety of darunavir/r versus lopinavir/r in treatment-experienced patients in TITAN. | Bánhegyi D, Katlama C, da Cunha CA, Schneider S, Rachlis A, Workman C, et al. | 2012 | DRV/r | Curr HIV Res |
| Efficacy and safety of raltegravir for treatment of HIV for 5 years in the BENCHMRK studies: final results of two randomised, placebo-controlled trials. | Eron JJ, Cooper DA, Steigbigel RT, Clotet B, Gatell JM, Kumar PN, et al. | 2013 | RAL | Lancet Infect Dis |
| **Studies presenting subgroup analyses** | | | | |
| Prognostic staging of extensively pretreated patients with advanced HIV-1 disease. | Montaner J, Guimaraes D, Chung J, Gafoor Z, Salgo M, DeMasi R. | 2005 | ENF | HIV Clin Trials |
| Food and Drug Administration analysis of tipranavir clinical resistance in HIV-1-infected treatment-experienced patients. | Naeger LK, Struble KA. | 2007 | TPV | AIDS |
| Subgroup and resistance analyses of raltegravir for resistant HIV-1 infection. | Cooper D a, Steigbigel RT, Gatell JM, Rockstroh JK, Katlama C, Yeni P, et al. | 2008 | RAL | N Engl J Med |
| Effect of baseline viral susceptibility on response to darunavir/ritonavir versus control protease inhibitors in treatment-experienced HIV type 1-infected patients: POWER 1 and 2. | Pozniak A, Opravil M, Beatty G, Hill A, de Béthune M-P, Lefebvre E. | 2008 | DRV/r | AIDS Res Hum Retroviruses |
| Adherence to enfuvirtide and its impact on treatment efficacy. | Rockstroh J, Dejesus E, Donatacci L, Wat C, Bertasso A, Labriola-Tompkins E, et al. | 2008 | ENF | AIDS Res Hum Retroviruses |
| Resistance profile of darunavir: combined 24-week results from the POWER trials. | de Meyer S, Vangeneugden T, van Baelen B, de Paepe E, van Marck H, Picchio G, et al. | 2008 | DRV/r | AIDS Res Hum Retroviruses |
| Efficacy of once-daily darunavir/ritonavir 800/100 mg in HIV-infected, treatment-experienced patients with no baseline resistance-associated mutations to darunavir. | De Meyer SMJ, Spinosa-Guzman S, Vangeneugden TJ, de Béthune M-P, Miralles GD. | 2008 | DRV/r | J Acquir Immune Defic Syndr |
| Subgroup analyses of maraviroc in previously treated R5 HIV-1 infection. | Fätkenheuer G, Nelson M, Lazzarin A, Konourina I, Hoepelman AI, Lampiris H, et al. | 2008 | MVC | N Engl J Med |
| Characterization of virologic failure patients on darunavir/ritonavir in treatment-experienced patients. | De Meyer S, Lathouwers E, Dierynck I, De Paepe E, Van Baelen B, Vangeneugden T, et al. | 2009 | DRV/r | AIDS |
| Identification of new genotypic cut-off levels to predict the efficacy of lopinavir/ritonavir and darunavir/ritonavir in the TITAN trial. | Hill A, Marcelin AG, Calvez V. | 2009 | DRV/r | HIV Med |
| Resistance profile of etravirine: combined analysis of baseline genotypic and phenotypic data from the randomized, controlled Phase III clinical studies. | Vingerhoets J, Tambuyzer L, Azijn H, Hoogstoel A, Nijs S, Peeters M, et al. | 2010 | ETR | AIDS |
| Characterization of genotypic and phenotypic changes in HIV-1-infected patients with virologic failure on an etravirine-containing regimen in the DUET-1 and DUET-2 clinical studies. | Tambuyzer L, Vingerhoets J, Azijn H, Daems B, Nijs S, de Béthune MP, et al. | 2010 | ETR | AIDS Res Hum Retroviruses |
| CD4+ T-cell restoration after 48 weeks in the maraviroc treatment-experienced trials MOTIVATE 1 and 2. | Asmuth DM, Goodrich J, Cooper DA, Haubrich R, Rajicic N, Hirschel B, et al. | 2010 | MVC | J Acquir Immune Defic Syndr |
| Impact of the background regimen on virologic response to etravirine: pooled 48-week analysis of DUET-1 and -2. | Trottier B, Di Perri G, Madruga J V, Peeters M, Vingerhoets J, Picchio G, et al. | 2010 | ETR | HIV Clin Trials |
| Impact of baseline antiretroviral resistance status on efficacy outcomes among patients receiving maraviroc plus optimized background therapy in the MOTIVATE 1 and 2 trials. | Nelson M, Fisher M, Gonzalez-Garcia J, Rockstroh JK, Weinstein D, Valdez H, et al. | 2010 | MVC | HIV Clin Trials |

ENF – enfuvirtide; TPV – tipranavir; /r -ritonavir; DRV – darunavir; ETR – etravirine; RAL – raltegravir; MVC – maraviroc;
