# Supplementary material for: A systematic review and meta-analysis assessing antiretroviral therapy for treatment-experienced HIV adult patients using an optimized background therapy approach: is there evidence enough for a standardized third-line strategy?
Source: Syst Rev. 2022 Nov 17;11:243. doi: 10.1186/s13643-022-02102-3 (PMC9673282; doi:10.1186/s13643-022-02102-3)
Supplement: Supplementary file 4 — Additional file 4. Search strategy used. [file 13643_2022_2102_MOESM4_ESM.docx]

**APPENDIX** - **Search strategy used**

1. MEDLINE (accessed by PubMed)

| [#17](http://www.ncbi.nlm.nih.gov/pubmed/?querykey=17&dbase=pubmed&querytype=eSearch&) | Search (#16) AND #11 |
| --- | --- |
| [#16](http://www.ncbi.nlm.nih.gov/pubmed/?querykey=16&dbase=pubmed&querytype=eSearch&) | Search (#14) AND #15 |
| [#15](http://www.ncbi.nlm.nih.gov/pubmed/?querykey=15&dbase=pubmed&querytype=eSearch&) | Search ((#8) OR #9) OR #10 |
| [#14](http://www.ncbi.nlm.nih.gov/pubmed/?querykey=14&dbase=pubmed&querytype=eSearch&) | Search (#12) AND #13 |
| [#13](http://www.ncbi.nlm.nih.gov/pubmed/?querykey=13&dbase=pubmed&querytype=eSearch&) | Search ((#5) OR #6) OR #7 |
| [#12](http://www.ncbi.nlm.nih.gov/pubmed/?querykey=12&dbase=pubmed&querytype=eSearch&) | Search (((#1) OR #2) OR #3) OR #4 |
| [#11](http://www.ncbi.nlm.nih.gov/pubmed/?querykey=11&dbase=pubmed&querytype=eSearch&) | Search randomized controlled trial [pt] OR controlled clinical trial [pt] OR randomized controlled trials [mh] OR random allocation [mh] OR double-blind method [mh] OR single-blind method [mh] OR clinical trial [pt] OR clinical trials[mh] OR (“clinical trial”[tw]) OR ((singl*[tw] OR doubl*[tw] OR trebl*[tw] OR tripl*[tw]) AND (mask*[tw] OR blind*[tw])) OR (placebos [mh] OR placebo* [tw] OR random* [tw] OR research design [mh:noexp] OR comparative study [pt] OR evaluation studies as topic [mh] OR follow-up studies [mh] OR prospective studies [mh] OR control* [tw] OR prospective* [tw] OR volunteer* [tw]) NOT (animals [mh] NOT humans [mh]) |
| [#10](http://www.ncbi.nlm.nih.gov/pubmed/?querykey=10&dbase=pubmed&querytype=eSearch&) | Search ("rescue therapy") OR ("rescue treatment") OR ("therapeutic failure”) |
| [#9](http://www.ncbi.nlm.nih.gov/pubmed/?querykey=9&dbase=pubmed&querytype=eSearch&) | Search "Drug Resistance"[Mesh]OR(Resistance, Drug) |
| [#8](http://www.ncbi.nlm.nih.gov/pubmed/?querykey=8&dbase=pubmed&querytype=eSearch&) | Search "Treatment Failure"[Mesh]OR(Failure, Treatment)OR(Failures, Treatment)OR(Treatment Failures) |
| [#7](http://www.ncbi.nlm.nih.gov/pubmed/?querykey=7&dbase=pubmed&querytype=eSearch&) | Search "Antiviral Agents"[Mesh:NoExp] OR (Agents, Antiviral )OR(Antiviral Drugs )OR(Drugs, Antiviral )OR(Antivirals) |
| [#6](http://www.ncbi.nlm.nih.gov/pubmed/?querykey=6&dbase=pubmed&querytype=eSearch&) | Search "Anti-Retroviral Agents"[Mesh]OR(Agents, Anti-Retroviral)OR(Anti Retroviral Agents)OR(Antiretroviral Agents)OR(Agents, Antiretroviral) |
| [#5](http://www.ncbi.nlm.nih.gov/pubmed/?querykey=5&dbase=pubmed&querytype=eSearch&) | Search Antiretroviral Therapy, Highly Active"[Mesh]OR(Highly Active Antiretroviral Therapy )OR(HAART) |
| [#4](http://www.ncbi.nlm.nih.gov/pubmed/?querykey=4&dbase=pubmed&querytype=eSearch&) | Search "Acquired Immunodeficiency Syndrome"[Mesh]OR(Immunologic Deficiency Syndrome, Acquired)OR(Acquired Immune Deficiency Syndrome)OR(Acquired Immuno-Deficiency Syndrome)OR(Acquired Immuno Deficiency Syndrome)OR(Acquired Immuno-Deficiency Syndromes)OR(Immuno-Deficiency Syndrome, Acquired)OR(Immuno-Deficiency Syndromes, Acquired)OR(Syndrome, Acquired Immuno-Deficiency)OR(Syndromes, Acquired Immuno-Deficiency)OR(Immunodeficiency Syndrome, Acquired)OR(Acquired Immunodeficiency Syndromes)OR(Immunodeficiency Syndromes, Acquired)OR(Syndrome, Acquired Immunodeficiency)OR(Syndromes, Acquired Immunodeficiency)OR(AIDS) |
| [#3](http://www.ncbi.nlm.nih.gov/pubmed/?querykey=3&dbase=pubmed&querytype=eSearch&) | Search "HIV-1"[Mesh]OR(Immunodeficiency Virus Type 1, Human)OR(Human Immunodeficiency Virus Type 1)OR(HIV-I)OR(Human immunodeficiency virus 1) |
| [#2](http://www.ncbi.nlm.nih.gov/pubmed/?querykey=2&dbase=pubmed&querytype=eSearch&) | Search "HIV Infections"[Mesh]OR(HIV Infection)OR(Infection, HIV)OR(Infections, HIV)OR(HTLV-III-LAV Infections)OR(HTLV III LAV Infections)OR(HTLV-III-LAV Infection)OR(Infection, HTLV-III-LAV)OR(Infections, HTLV-III-LAV)OR(T-Lymphotropic Virus Type III Infections, Human)OR(T Lymphotropic Virus Type III Infections, Human)OR(HTLV-III Infections)OR(HTLV III Infections)OR(HTLV-III Infection)OR(Infection, HTLV-III)OR(Infections, HTLV-III) |
| [#1](http://www.ncbi.nlm.nih.gov/pubmed/?querykey=1&dbase=pubmed&querytype=eSearch&) | Search "HIV"[Mesh]OR(Viruses, Human Immunodeficiency)OR(AIDS Virus)OR(AIDS Viruses)OR(Virus, AIDS)OR(Viruses, AIDS)OR(HTLV-III)OR(Human Immunodeficiency Virus)OR(Human Immunodeficiency Viruses)OR(Human T Cell Lymphotropic Virus Type III)OR(Human T Lymphotropic Virus Type III)OR(Human T-Cell Leukemia Virus Type III)OR(Human T Cell Leukemia Virus Type III)OR(Human T-Cell Lymphotropic Virus Type III)OR(Human T-Lymphotropic Virus Type III)OR(Immunodeficiency Virus, Human)OR(Immunodeficiency Viruses, Human)OR(LAV-HTLV-III)OR(Lymphadenopathy-Associated Virus)OR(Lymphadenopathy Associated Virus)OR(Lymphadenopathy-Associated Viruses)OR(Virus, Lymphadenopathy-Associated)OR(Viruses, Lymphadenopathy-Associated)OR(Virus, Human Immunodeficiency)OR(Acquired Immune Deficiency Syndrome Virus)OR(Acquired Immunodeficiency Syndrome Virus) |

1. EMBASE

| #19 #16 AND #18 |  |  |
| --- | --- | --- |
| #18 'randomized controlled trial'/exp OR 'controlled clinical trial'/exp OR 'randomization'/exp OR 'double blind procedure'/exp OR 'single blind procedure'/exp OR 'clinical trial'/exp OR 'clinical trial'/syn OR (singl* OR doubl* OR trebl* OR tripl* AND (mask* OR 'blind'/syn)) OR 'placebo'/exp OR placebo* OR random* OR 'crossover procedure'/syn NOT ('animal'/exp NOT 'human'/exp) AND [embase]/lim |  |  |
| #16 #14 AND #15 |  |  |
| #15 #12 AND #13 |  |  |
| #14 #8 OR #9 OR #10 |  |  |
| #13 #5 OR #6 OR #7 |  |  |
| #12 #1 OR #2 OR #3 OR #4 |  |  |
| #10 'rescue therapy' OR 'rescue treatment' OR 'therapeutic failure' AND [embase]/lim |  |  |
| #9 'drug resistance'/exp AND [embase]/lim |  |  |
| #8 'treatment failure'/exp AND [embase]/lim |  |  |
| #7 'antivirus agent'/exp AND [embase]/lim |  |  |
| #6 'antiretrovirus agent'/exp AND [embase]/lim |  |  |
| #5 'highly active antiretroviral therapy'/exp AND [embase]/lim |  |  |
| #4 'acquired immune deficiency syndrome'/exp AND [embase]/lim |  |  |
| #3 'human immunodeficiency virus 1'/exp AND [embase]/lim |  |  |
| #2 'human immunodeficiency virus infection'/exp AND [embase]/lim |  |  |
| #1 'human immunodeficiency virus'/exp AND [embase]/lim |  |  |

1. SCOPUS

| 17 | #16 AND #11 |
| --- | --- |
| 16 | #14 AND #15 |
| 15 | #8 OR #9 OR #10 |
| 14 | #12 AND #13 |
| 13 | #5 OR #6 OR #7 |
| 12 | #1 OR #2 OR #3 OR #4 |
| 11 | ((TITLE-ABS-KEY("randomized controlled trial") OR TITLE-ABS-KEY("controlled clinical trial") OR TITLE-ABS-KEY("random allocation") OR TITLE-ABS-KEY("clinical trial") OR TITLE-ABS-KEY("placebo"))) OR (TITLE-ABS-KEY(random*)) |
| 10 | TITLE-ABS-KEY(("rescue therapy") OR ("rescue treatment") OR ("therapeutic failure")) |
| 9 | INDEXTERMS("Drug Resistance") OR TITLE-ABS-KEY(resistance, drug) |
| 8 | INDEXTERMS("Treatment Failure") OR TITLE-ABS-KEY((failure, treatment) OR (failures, treatment) OR (treatment failures)) |
| 7 | INDEXTERMS("Antiviral Agents") OR TITLE-ABS-KEY((agents, antiviral) OR (antiviral drugs) OR (drugs, antiviral) OR (antivirals)) |
| 6 | INDEXTERMS("Anti-Retroviral Agents") OR TITLE-ABS-KEY((agents, anti-retroviral) OR (anti retroviral agents) OR (antiretroviral agents) OR (agents, antiretroviral)) |
| 5 | INDEXTERMS("Antiretroviral Therapy, Highly Active") OR TITLE-ABS-KEY((highly active antiretroviral therapy) OR (haart)) |
| 4 | INDEXTERMS("Acquired Immunodeficiency Syndrome") OR TITLE-ABS-KEY((immunologic deficiency syndrome, acquired) OR (acquired immune deficiency syndrome) OR (acquired immuno-deficiency syndrome) OR (acquired immuno deficiency syndrome) OR (acquired immuno-deficiency syndromes) OR (immuno-deficiency syndrome, acquired) OR (immuno-deficiency syndromes, acquired) OR (syndrome, acquired immuno-deficiency) OR (syndromes, acquired immuno-deficiency) OR (immunodeficiency syndrome, acquired) OR (acquired immunodeficiency syndromes) OR (immunodeficiency syndromes, acquired) OR (syndrome, acquired immunodeficiency) OR (syndromes, acquired immunodeficiency) OR (aids)) |
| 3 | INDEXTERMS("HIV-1") OR TITLE-ABS-KEY((immunodeficiency virus TYPE 1, human) OR (human immunodeficiency virus TYPE 1) OR (hiv-i) OR (human immunodeficiency virus 1)) |
| 2 | INDEXTERMS("HIV Infections") OR TITLE-ABS-KEY((hiv infection) OR (infection, hiv) OR (infections, hiv) OR (htlv-iii-lav infections) OR (htlv iii lav infections) OR (htlv-iii-lav infection) OR (infection, htlv-iii-lav) OR (infections, htlv-iii-lav) OR (t-lymphotropic virus TYPE iii infections, human) OR (t lymphotropic virus TYPE iii infections, human) OR (htlv-iii infections) OR (htlv iii infections) OR (htlv-iii infection) OR (infection, htlv-iii) OR (infections, htlv-iii)) |
| 1 | INDEXTERMS("HIV") OR TITLE-ABS-KEY((viruses, human immunodeficiency) OR (aids virus) OR (aids viruses) OR (virus, aids) OR (viruses, aids) OR (htlv-iii) OR (human immunodeficiency virus) OR (human immunodeficiency viruses) OR (human t cell lymphotropic virus TYPE iii) OR (human t lymphotropic virus TYPE iii) OR (human t-cell leukemia virus TYPE iii) OR (human t cell leukemia virus TYPE iii) OR (human t-cell lymphotropic virus TYPE iii) OR (human t-lymphotropic virus TYPE iii) OR (immunodeficiency virus, human) OR (immunodeficiency viruses, human) OR (lav-htlv-iii) OR (lymphadenopathy-associated virus) OR (lymphadenopathy associated virus) OR (lymphadenopathy-associated viruses) OR (virus, lymphadenopathy-associated) OR (viruses, lymphadenopathy-associated) OR (virus, human immunodeficiency) OR (acquired immune deficiency syndrome virus) OR (acquired immunodeficiency syndrome virus)) |

1. ISI Web of Science

| # 17 | #16 AND #11  *Databases=SCI-EXPANDED, SSCI, A&HCI Timespan=All Years* |
| --- | --- |
| # 16 | #15 AND #14  *Databases=SCI-EXPANDED, SSCI, A&HCI Timespan=All Years* |
| # 15 | #10 OR #9 OR #8  *Databases=SCI-EXPANDED, SSCI, A&HCI Timespan=All Years* |
| # 14 | #13 AND #12  *Databases=SCI-EXPANDED, SSCI, A&HCI Timespan=All Years* |
| # 13 | #7 OR #6 OR #5  *Databases=SCI-EXPANDED, SSCI, A&HCI Timespan=All Years* |
| # 12 | #4 OR #3 OR #2 OR #1  *Databases=SCI-EXPANDED, SSCI, A&HCI Timespan=All Years* |
| # 11 | TS= ("randomized controlled trial" OR "controlled clinical trial" OR "randomized controlled trials" OR "random allocation" OR "double-blind method" OR "single-blind method" OR "clinical trial" OR "clinical trials" OR "clinical trial" OR "placebos" OR placebo* OR random* OR "research design" OR "comparative study" OR "evaluation studies as topic" OR "follow-up studies" OR "prospective studies" OR control* OR prospective* OR volunteer*) OR TS= ((singl* OR doubl* OR trebl* OR tripl*) AND (mask* OR blind*))  *Databases=SCI-EXPANDED, SSCI, A&HCI Timespan=All Years* |
| # 10 | TS= ("Rescue Therapy" OR "Rescue Treatment" OR "Therapeutic Failure")  *Databases=SCI-EXPANDED, SSCI, A&HCI Timespan=All Years* |
| # 9 | TS= ("Drug Resistance" OR "Resistance, Drug")  *Databases=SCI-EXPANDED, SSCI, A&HCI Timespan=All Years* |
| # 8 | TS= ("Treatment Failure" OR "Failure, Treatment" OR "Failures, Treatment" OR "Treatment Failures")  *Databases=SCI-EXPANDED, SSCI, A&HCI Timespan=All Years* |
| # 7 | TS= ("Antiviral Agents" OR "Agents, Antiviral" OR "Antiviral Drugs" OR "Drugs, Antiviral" OR "Antivirals")  *Databases=SCI-EXPANDED, SSCI, A&HCI Timespan=All Years* |
| # 6 | TS= ("Anti-Retroviral Agents" OR "Agents, Anti-Retroviral" OR "Anti Retroviral Agents" OR "Antiretroviral Agents" OR "Agents, Antiretroviral")  *Databases=SCI-EXPANDED, SSCI, A&HCI Timespan=All Years* |
| # 5 | TS= ("Antiretroviral Therapy, Highly Active" OR "Highly Active Antiretroviral Therapy" OR "HAART")  *Databases=SCI-EXPANDED, SSCI, A&HCI Timespan=All Years* |
| # 4 | TS= ("Acquired Immunodeficiency Syndrome" OR "Immunologic Deficiency Syndrome, Acquired" OR "Acquired Immune Deficiency Syndrome" OR "Acquired Immuno-Deficiency Syndrome" OR "Acquired Immuno Deficiency Syndrome" OR "Acquired Immuno-Deficiency Syndromes" OR "Immuno-Deficiency Syndrome, Acquired" OR "Immuno-Deficiency Syndromes, Acquired" OR "Syndrome, Acquired Immuno-Deficiency" OR "Syndromes, Acquired Immuno-Deficiency" OR "Immunodeficiency Syndrome, Acquired" OR "Acquired Immunodeficiency Syndromes" OR "Immunodeficiency Syndromes, Acquired" OR "Syndrome, Acquired Immunodeficiency" OR "Syndromes, Acquired Immunodeficiency" OR "AIDS")  *Databases=SCI-EXPANDED, SSCI, A&HCI Timespan=All Years* |
| # 3 | TS= ("HIV-1" OR "Immunodeficiency Virus Type 1, Human" OR "Human Immunodeficiency Virus Type 1" OR "HIV-I" OR "Human immunodeficiency virus 1")  *Databases=SCI-EXPANDED, SSCI, A&HCI Timespan=All Years* |
| # 2 | TS= ("HIV Infections" OR "HIV Infection" OR "Infection, HIV" OR "Infections, HIV" OR "HTLV-III-LAV Infections" OR "HTLV III LAV Infections" OR "HTLV-III-LAV Infection" OR "Infection, HTLV-III-LAV" OR "Infections, HTLV-III-LAV" OR "T-Lymphotropic Virus Type III Infections, Human" OR "T Lymphotropic Virus Type III Infections, Human" OR "HTLV-III Infections" OR "HTLV III Infections" OR "HTLV-III Infection" OR" Infection, HTLV-III" OR "Infections, HTLV-III")  *Databases=SCI-EXPANDED, SSCI, A&HCI Timespan=All Years* |
| # 1 | TS= ("HIV" OR "Viruses, Human Immunodeficiency" OR "AIDS Virus" OR "AIDS Viruses" OR "Virus, AIDS" OR "Viruses, AIDS" OR "HTLV-III" OR "Human Immunodeficiency Virus" OR "Human Immunodeficiency Viruses" OR "Human T Cell Lymphotropic Virus Type III" OR "Human T Lymphotropic Virus Type III" OR "Human T-Cell Leukemia Virus Type III" OR "Human T Cell Leukemia Virus Type III" OR "Human T-Cell Lymphotropic Virus Type III" OR "Human T-Lymphotropic Virus Type III" OR "Immunodeficiency Virus, Human" OR "Immunodeficiency Viruses, Human" OR "LAV-HTLV-III" OR "Lymphadenopathy-Associated Virus" OR "Lymphadenopathy Associated Virus" OR "Lymphadenopathy-Associated Viruses" OR "Virus, Lymphadenopathy-Associated" OR "Viruses, Lymphadenopathy-Associated" OR "Virus, Human Immunodeficiency" OR "Acquired Immune Deficiency Syndrome Virus" OR "Acquired Immunodeficiency Syndrome Virus")  *Databases=SCI-EXPANDED, SSCI, A&HCI Timespan=All Years* |

1. Cochrane Central Register of Controlled Trials

| #14 | [(#11 AND #12 AND #13)](http://onlinelibrary.wiley.com/o/cochrane/searchHistory?mode=runquery&qnum=14) |
| --- | --- |
| #13 | [(#8 OR #9 OR #10)](http://onlinelibrary.wiley.com/o/cochrane/searchHistory?mode=runquery&qnum=13) |
| #12 | [(#5 OR #6 OR #7)](http://onlinelibrary.wiley.com/o/cochrane/searchHistory?mode=runquery&qnum=12) |
| #11 | [(#1 OR #2 OR #3 OR #4)](http://onlinelibrary.wiley.com/o/cochrane/searchHistory?mode=runquery&qnum=11) |
| #10 | [("rescue therapy") OR ("rescue treatment") OR ("therapeutic failure"):ti,ab,kw](http://onlinelibrary.wiley.com/o/cochrane/searchHistory?mode=runquery&qnum=10) |
| #9 | [MeSH descriptor **Drug Resistance** explode all trees](http://onlinelibrary.wiley.com/o/cochrane/searchHistory?mode=runquery&qnum=9) |
| #8 | [MeSH descriptor **Treatment Failure** explode all trees](http://onlinelibrary.wiley.com/o/cochrane/searchHistory?mode=runquery&qnum=8) |
| #7 | [MeSH descriptor **Antiviral Agents**, this term only](http://onlinelibrary.wiley.com/o/cochrane/searchHistory?mode=runquery&qnum=7) |
| #6 | [MeSH descriptor **Anti-Retroviral Agents** explode all trees](http://onlinelibrary.wiley.com/o/cochrane/searchHistory?mode=runquery&qnum=6) |
| #5 | [MeSH descriptor **Antiretroviral Therapy, Highly Active** explode all trees](http://onlinelibrary.wiley.com/o/cochrane/searchHistory?mode=runquery&qnum=5) |
| #4 | [MeSH descriptor **Acquired Immunodeficiency Syndrome** explode all trees](http://onlinelibrary.wiley.com/o/cochrane/searchHistory?mode=runquery&qnum=4) |
| #3 | [MeSH descriptor **HIV-1** explode all trees](http://onlinelibrary.wiley.com/o/cochrane/searchHistory?mode=runquery&qnum=3) |
| #2 | [MeSH descriptor **HIV Infections** explode all trees](http://onlinelibrary.wiley.com/o/cochrane/searchHistory?mode=runquery&qnum=2) |
| #1 | [MeSH descriptor **HIV** explode all trees](http://onlinelibrary.wiley.com/o/cochrane/searchHistory?mode=runquery&qnum=1) |

1. LILACS

| #3 | #1 AND #2 |
| --- | --- |
| #2 | ("anti-HIV agents" OR "agents anti VIH" OR "Agentes anti-HIV" OR "Antiretroviral therapy highly active" OR "TARV altamente activa" OR "Terapia anti-retroviral de alta atividade" OR HAART OR "Antiviral agents" OR "Agentes antivirales" OR Antivirais OR "Agentes antivirais") |
| #1 | (HIV OR VIH OR "HIV Infections" OR Infecciones por HIV" OR "infecções por HIV" OR "HIV-1 OR "VIH-1" OR "Acquired immunodeficiency syndrome" OR sindrome de imunodeficiencia adquirida" OR AIDS OR SIDA) |
